# Critical Role of Kupffer Cell CD89 Expression in Experimental IgA Nephropathy

**DOI:** 10.1371/journal.pone.0159426

**Published:** 2016-07-20

**Authors:** Lijun Xu, Bingyu Li, Mengwen Huang, Kun Xie, Dong Li, You Li, Hua Gu, Jianmin Fang

**Affiliations:** 1 School of Life Sciences and Technology, Tongji University, Shanghai, China; 2 Shanghai Tongji Hospital, Tongji University, Shanghai, China; 3 Tongji University Suzhou Institute, Suzhou, Jiangsu, China; 4 Collaborative Innovation Center for Biotherapy, West China Hospital, Sichuan University, Chengdu, Sichuan, China; Radboud university medical center, NETHERLANDS

## Abstract

Although IgA nephropathy (IgAN) is the most common primary glomerulonephritis worldwide, its etiology remains only partly understood. It is clear that the pathogenesis of IgAN involves the formation of macromolecular IgA1 complexes and increased levels of serum IgA1 and IgA1-immune complexes(IC), due to defective IgA1 clearance. Previous studies suggest that the blood and tissue myeloid cell-expressed IgA Fc receptor (FcαR/CD89) mediates IgA-IC clearance and its dysfunction, via decreased activity or excessive levels of soluble FcαR/sCD89 induces IgAN. Such a mechanism requires robust stimulation of IgAN levels via forced expression of CD89. In the absence of unequivocal evidence supporting such a mechanism to date, we attempted to test the extent of CD89-evoked IgAN by generating a transgenic mouse strain expressing human CD89 under the control of murine CD14 promotor. No deposition of IgA-CD89 complexes or glomerulonephritis was detected, however. Further studies showed that elimination of murine IgA was mediated by Kupffer cells. In patients, however, CD89/IgA complexes were detected, and injection of patient IgA induced IgAN-like features in CD89 Tg mice. In transgenic mice, IgAN pathogenesis involves impaired clearance of abnormal IgA via CD89, primarily by the Kupffer cells. Conditional IgAN progression in CD89 transgenic mice thus reveals important aspects of IgAN pathogenesis.

## Introduction

IgA nephropathy (IgAN) is a major cause of renal failure[1,2]. A defining feature of the disease is the presence of mesangial IgA deposits, usually containing IgA1[3]. Central to the pathogenesis of IgAN is the formation of circulating IgA immune complexes (IgA-ICs) that are deposited in the renal mesangial areas, triggering glomerular injury. Various components of IgA-ICs include C3, IgG, IgM and fibronectin[4–6]. Increased serum levels of IgA1 and IgA1-IC were observed in patients with IgAN[7,8] and appear at least partly derived from overproduction of IgA1 by B cells[9]. However, impaired clearance of IgA1 and IgA1-IC by dysfunctional IgA receptors has also been reported. Aberrant IgA glycosylation is believed to generate *de novo* antigenic epitopes that are subsequently recognized by naturally occurring IgG and IgA1, leading to the formation of circulating immune complexes[10–12]. Remarkably, such abnormally glycosylated IgA is unlikely to be recognized by the asialoglycoprotein receptor (ASGPR) on hepatocytes and therefore escapes hepatic clearance[13,14].

In addition to the ASGPR, the Fc-α receptor (CD89) is also a principal component underlying IgA catabolism and clearance of IgA-ICs from the circulation. CD89 is a protein expressed by human monocytes/macrophages (including Kupffer cells), neutrophils, eosinophils and dendritic cells, and acts as a specific receptor for IgA[15–17]. Binding of IgA and IgA-IC to CD89 triggers macrophage activation and IgA elimination[18]. In IgAN patients, decreased expression of CD89 was detected in myeloid cells despite normal levels of transcription, and delayed kinetics of CD89-mediated endocytosis[19,20]. Nevertheless, the exact functionality of CD89 remains controversial and requires further investigation.

Previous work suggested that CD89 regulates IgAN expression via formation of sCD89-IgA complexes. Following IgA-dependent shedding, the protein enters the circulation to form sCD89. Complicating interpretation of the role of CD89 in IgAN is the existence of a smaller, only marginally glycosylated 30-kDa sCD89 isoform. Transgenic mice overexpressing human CD89 spontaneously developed massive mesangial IgA deposition, glomerular and interstitial macrophage infiltration, mesangial matrix expansion, hematuria, and mild proteinuria apparently providing equivocal evidence for a key role of soluble CD89 in the pathogenesis of IgAN[21]. Later, however, it emerged that in these experiments a human CD11b promoter and not a murine CD11b promoter drove transgene expression. Therefore, experiments in transgenic mice employing a genuine murine phagocyte promotor are needed to elucidate the role of CD89 in IgA clearance and sCD89-IgA complex formation in IgAN,

In this study, we generated a CD89 transgenic mouse strain using an authentic murine CD14 promotor with CD89 expression on blood and tissue monocytes/macrophages. These animals, however, showed no signs of IgAN but displayed concomitant increased clearance of mouse serum IgA. In these experiments Kupffer cells were the predominant cell type in the degradation of experimentally-induced human IgA, especially human IgA1 catabolized by these cells. Injecting large quantities of purified IgA from IgAN patients into CD89 Tg mice triggers IgAN, and concomitant dysfunctional endocytosis and elimination of patient IgA, resulting in the deposition of CD89-IgA in kidneys of Tg mice. Our results indicate that although CD89 plays an important role in IgA and IgA-IC clearance, defective endocytosis of underglycosylated IgA is necessary for the development of experimental IgAN.

## Materials and Methods

### Ethics Statement

The study was performed according to the Declaration of Helsinki and approved by the Institutional Ethics Committee of Tongji Hospital in Shanghai, China. Our study was reviewed and monitored by Dr Lingjing Jin, member of the Institutional Ethics Committee of Tongji Hospital. Written informed consent was obtained from the patients. All participants were over the age of 18.

All animal experiments were approved by the Animal Ethics Committee of Tongji University and were in accordance with the Association for Research in Vision and Ophthalmology (ARVO) statement for the use of Animals in Ophthalmic and Vision Research. C57BL/6 mice were housed in a pathogen-free animal facility at the experimental animal center of Tongji University. Mice were fed standard chow and provided with distilled water *ad libitum*. Fresh cages were provided weekly. Pentobarbital sodium (60 mg/kg) was used as the anesthetic drug, and mice were euthanized using 100 mg/kg sodium pentobarbital. Appropriate efforts were made to minimize animal suffering.

### Human Subjects and IgA Preparation

In order to determine human IgA clearance via CD89, human IgA was purified from healthy human serum or IgAN patient serum using Jacalin/Agarose (Life Technologies, Carlsbad, CA, USA) according to the manufacturer’s instructions. Sera were obtained from 10 unrelated patients with biopsy-proven IgAN (5 males and 5 females), and from 10 healthy donors (5 males and 5 females). None of the patients displayed clinical evidence of Henoch–Schönlein purpura, systemic lupus erythematosus or liver disease, or received immunosuppressive therapy. Venous blood and urine samples were collected and clinical characteristics of the patient group were obtained (S1 Table). From all patients, clinical and laboratory data, such as the estimated glomerular filtration rate (GFR) using the Cockcroft–Gault formula, were obtained retrospectively. Laboratory findings also indicated a higher serum IgA, creatinine and BUN levels.

### Transgenic Mice

The generation of CD89 transgenic mice was performed with the assistance of the Shanghai Biomodel Organism Science & Technology Development Co.Ltd., Shanghai, China. We designed a CD14-targeting vector containing the human CD89 and homologous arms (2.6kb) on both the 5’ and the 3’ ends, targeting the CD89 gene to the murine CD14 locus using homologous recombination, employing the 2A self-processing sequence[22] to achieve mouse CD14 promoter-controlled expression of CD89. Homologous recombinant ES cells were injected into C57BL/6 blastocysts, and transferred into the pseudopregnant females. The resulting chimeric male mice were mated to C57BL/6 females, and interbred heterozygous offspring were created. As the *frt-neo-frt* cassette may affect expression, the CD89 transgenic mice were mated with the CAG-*cre* transgenic mice to excise *loxP*-flanked *neo* cassette *in vivo*. Heterozygous mutant mice without the *neo* cassette were phenotypically normal, and these mice were intercrossed to breed homozygous mutant mice. The CD89 homozygous offspring were detected by PCR of tail DNA using transgene-specific primers (5’-GACGCAGAACTTGATCCGCA-3’ and 5’-GGATTCGTTGACGAGGACCC-3’).

The non-human CD89-gene element-containing littermates from the same heterozygous mice were used as controls. All mouse strains used in this study were raised and housed strictly under pathogen-free conditions. For all animal experimentation appropriate ethical permission was obtained.

### Flow Cytometry

Flow cytometry was used for identification of various immune cell types and CD89 expression was analyzed in different tissues of Tg mice. Monocytes were defined as Ly-6C^+^ subsets; lymphocytes as CD45/B220^+^; and neutrophils as Gr-1^+^. The antibodies mentioned above were purchased from BD PharMingen, San Diego, CA. FITC-conjugated anti-CD89 antibody (CD89-FITC) (Miltenyi Biotech, Bergisch Gladbach, Germany) was used to determine surface expression of hFcαRI. Cells (10^6^) from the bone marrow, heparinized blood or peritoneal cavity were incubated with FITC-conjugated anti-CD89 antibody and its isotype for 30 min at 4°C.

Cells were resuspended in complete culture medium and kept in a water bath at 37°C for 60 min. and cells were stained for 30 min at 2–8°C. The cells were washed with 2 mL of FACS flow and spin out at 4°C. Antibodies were added and cells were stained for 30 min at 2–8°C. Cells were washed as described before and resuspended in 200 μL of FACS buffer for flow cytometric analysis. In robustness analysis, cells were resuspended in 200 μL of 1% formaldehyde and incubated for 24 h at 2–8°C before flow cytometric analysis.

### Detection of IgA-CD89 Interaction

To determine IgA binding by FACS, murine peritoneal macrophages were used. Cellular concentration was adjusted to 1 x 10^6^ cells/mL, of which 2 x 10^5^ cells were stained. Cells were incubated with mouse mIgA (Bethyl Laboratories, Montgomery, TX), mouse dimer IgA, human mIgA (Abcam, Cambridge, UK) and human secretory dIgA (Abd Serotec, Oxford, UK) for 1 h at 4°C, and supplemented with mAbs and their isotype antibodies according to the experimental protocol. The monoclonal mouse anti-human CD89 antibodies (MIP8a, Abcam), which blocks IgA binding, was used as a negative control. After washing, cells were incubated with FITC-labeled rat anti-mouse IgA and FITC-labeled rat anti-human IgA (BD Pharmingen, San Diego, CA), respectively, for 30 min at 4°C. Cells were analyzed on a FACSVerse (BD Pharmingen).

To analyze the binding of human and mouse IgA to human CD89, ELISAs were used. Serial dilutions of human or mouse mIgA and dIgA were coated into 96-well plates overnight. Wells were washed and blocked with 1% BSA in PBS, and 100 μL of purified recombinant human CD89 (20 μg/mL, Sino Biological, Beijing, China) was added. After 2 h of incubation at 37°C, plates were washed and incubated with rabbit polyclonal IgG anti-human CD89 conjugated with HRP (diluted 1:2000; Lifespan biosciences), as appropriate, for 1 h at 37°C. After washing, the reaction was developed with HRP substrate, and the reaction was terminated with 2M H_2_SO_4_. Absorbance was read at 405 nm in a Multiscan MS spectrophotometer (Labsystems, Helsinki, Finland).

### ELISA

To identify sCD89-IgA complex in the cell supernatant after IgA and CD89 binding, 96-well plates were coated overnight with mouse anti-human CD89 (A3; Beckman Coulter) at a concentration of 1 μg/well, washed and blocked with 1% BSA in PBS. The cell supernatant was diluted in PBST-1% BSA diluted 1:2000. The internal control included 1 μg/mL human IgA binding to excess recombinant CD89 (5 μg/mL; Sino Biological, Beijing, China). After 2 h of incubation at 37°C, plates were washed and mouse anti-human IgA-conjugated HRP (diluted 1:5000; Abcam) was added and incubated for 1h at 37°C. After washing, the reaction was developed with HRP substrate, and the reaction was terminated with 2M H_2_SO_4_. Absorbance was read at 405 nm in a Multiscan MS spectrophotometer (Labsystems, Helsinki, Finland). The mouse IgA, IgG, and IgM or human IgA concentration in mouse serum were determined using mouse IgA, IgG, IgM or human IgA ELISA Kits (Bethyl lab), respectively, according to the manufacturer’s instructions.

### Western Blot

Western blot was performed as described [23]. The lysate from different tissues of CD89 transgenic mice and littermate WT mice was diluted in phosphate-buffered saline (PBS) and mixed with reducing sample buffer (Life Technologies, Carlsbad, CA, USA), and boiled for 9 min. 15 μL of serum (diluted 1:20) and tissue lysate (30 μg per well) were loaded on the gels, using the broad range protein molecular weight marker (Life Technologies) as protein size indicator. Mouse anti-human CD89 antibody A59 (BD Pharmingen) was used as primary antibody. HRP-conjugated goat anti-mouse IgG (Bethyl Laboratories) was used as secondary antibody. To detect the IgA levels produced by B lymphocytes in the blood, goat anti-mouse IgA antibody and HRP-conjugated rat anti-goat IgG antibody (Abcam) were used.

### Determination of Intracellular Reactive Oxygen Species (ROS)

Bone marrow-derived macrophages (BMDM) were isolated and cultured as described [24]. To stimulate BMDM, 10^6^ cells were incubated for 30 min at room temperature with 10 μg/mL F(ab′)_2_ fragments of a MIP8a antibody, generated through digestion with pepsin (Sigma-Aldrich). After washing twice with RPMI 1640, goat F(ab’)_2_ anti-mouse IgG (H+L) (SouthernBiotech, Birmingham, AL) was added for 2 h at 37°C to crosslink CD89. As positive controls 2 U/mL IFNγ (PeproTech, Rocky Hill, NJ) and 10 ng/mL LPS (Sigma-Aldrich, St Louis, MO) added to 10^6^ BMDM cells for 4 h at 37°C were used. The negative control lacked the cross-linking Ab. Subsequently, CD89-specific intracellular ROS production was measured by flow cytometry using a cell-based ROS assay kit (Beyotime Institute of Biotechnology, Shanghai, China) following the manufacturer’s instructions. Macrophages were trypsinized, washed with PBS, and then incubated with DCFH-DA at a final concentration of 10 μM for 30 min at 37°C. Fluorescence was analyzed on a FACSVerse (BD Pharmingen). Intracellular ROS levels were expressed as the average DCF fluorescence intensity of the cells.

### IL-2 Production

10^6^ BMDM cells were incubated for 30 min at 4°C, together with 10 μg/mL MIP8a-F(ab’)_2_. After washing with PBS, goat F(ab’)_2_ anti-mouse IgG (H+L) was added to the cells and incubated overnight at 37°C. As a negative control, cells were only incubated with the anti-CD89 mAb. The B cell receptor cross-linked with goat anti-mouse IgG (BD Pharmingen) served as a positive control. After overnight incubation, supernatants were isolated, and IL-2 production was measured by mouse IL-2 ELISA kit (R&D Systems, Minneaoplis, MN) as described before.

### Histology, Immunohistochemistry and Immunofluorescence

Paraffin-embedded kidney sections (4-μm-thick) were stained with hematoxylin and eosin stain (H&E) and PAS for morphological analysis. For immunohistochemistry, 3-4-μm-thick paraffin-embedded tissue sections were transferred to microscope slides, and incubated with primary antibodies rabbit anti-human CD89 antibody (Abcam) or goat anti-mouse IgA (Southern Biotech) for 1 h at room temperature in a humidified chamber. The stained mouse dedecadactylon IgA served as the positive control of anti-mouse IgA mAb (S1 Fig). If appropriate, incubation with the primary antibody was followed by incubation with biotinylated anti-rabbit IgG or anti-goat IgG (Beyotime). Subsequently, the avidin/biotin-horseradish peroxidase complex (ABC-HRP reagent; Beyotime) was added, followed by application of 3,3’-diaminobenzidine tetrahydrochloride (DAB), according to routine procedures. Slides were then counterstained with hematoxylin and mounted on coverslips.

To determine CD89 expression in frozen tissue sections of CD89 transgenic mice, sections were incubated successively with primary antibodies rabbit anti-human CD89, rat anti-mouse CD68 (Abcam) overnight at 4°C, followed by incubation with FITC-labeled goat anti-rabbit Ab or Cy3-labeled goat anti-rat Ab (Beyotime), all according to established procedures. Slides were probed with a laser-scanning confocal microscope (Nicon, Tokyo, Japan).

To assess the role of monocyte CD89/γ2 receptors for IgA clearance, FITC-labeled human IgA was injected into the tail vein of C57BL/6-Tg mice. At various time intervals following injection, mice were sacrificed and livers were stained with a rabbit anti–human CD89 mAb (Abcam) and a Cy3-goat anti-rabbit IgG (Beyotime) according to routine procedures. Slides were investigated using a laser-scanning confocal microscope (Nicon), employing routine methodology.

To detect the IgA clearance by Kupffer cells in CD89 transgenic mice we employed an *in vitro* assay. Isolation and culture of Kupffer cells was performed as described earlier[25]. Cy3-labeled human mIgA (Abcam, 1 μg/mL) was incubated with Kupffer cells from CD89 transgenic mice for 1 h at 37°C. Subsequently, cells were coverslipped, followed by fixation, permeabilization, staining, and confocal microscopy according to routine procedures.

### ADCC

The CytoTox96 non-radioactive assay (Promega, Madison, USA) was used to evaluate the capacity of mouse blood cells to trigger lysis of tumor cells. Monocytes were selected from peripheral blood mononuclear cells (PBMCs) of CD89 transgenic and WT mice using CD14-positive microbeads (Miltenyi Biotech, Bergisch Gladbach, Germany), and plated (5×10^4^/well) in 96-well plates. Raji cells (5×10^3^/well) were also transferred to the same plates and cultures were incubated with a recombinant anti-CD20 IgA antibody generated at our lab in different concentrations (0, 0.1, 1.0, 10.0 μg/mL) at 37°C for 20 h, an IgA isotype (Abcam) was used as a negative control. The lactate dehydrogenase (LDH) released into the medium following challenge with the anti-CD20 IgA was quantified by measuring absorbance at 490 nm. The LDH activity in supernatants from co-cultures of monocytes and Raji cells, monocytes cultured in the absence of cell lysis target, and Raji cells cultured in the absence of monocytes, was used to calculate corrected experimental, effector and target values, respectively. These values were then used to calculate cytotoxicity expressed as a percentage of the target cells according to routine procedures.

### Kidney Functional Parameters

For hematuria, 10 μL of fresh urine was used to count erythrocytes using a hemocytometer. The detection of blood urea nitrogen (BUN) levels in sera and urinary protein in urine samples of mice was carried out using diagnostic kits purchased from Nanjing Jiancheng Bioengineering Institute (Nanjing, China) according the guidelines provided by the manufacturer.

### Statistical Analysis

Differences between groups were determined using the Kruskal–Wallis Tests and the nonparametric Mann-Whitney U test, as indicated. A P <0.05 was considered significant.

## Results

### Human FcαRI Expression in the Monocytes/Macrophages of Transgenic Mice

**Fig 1:**
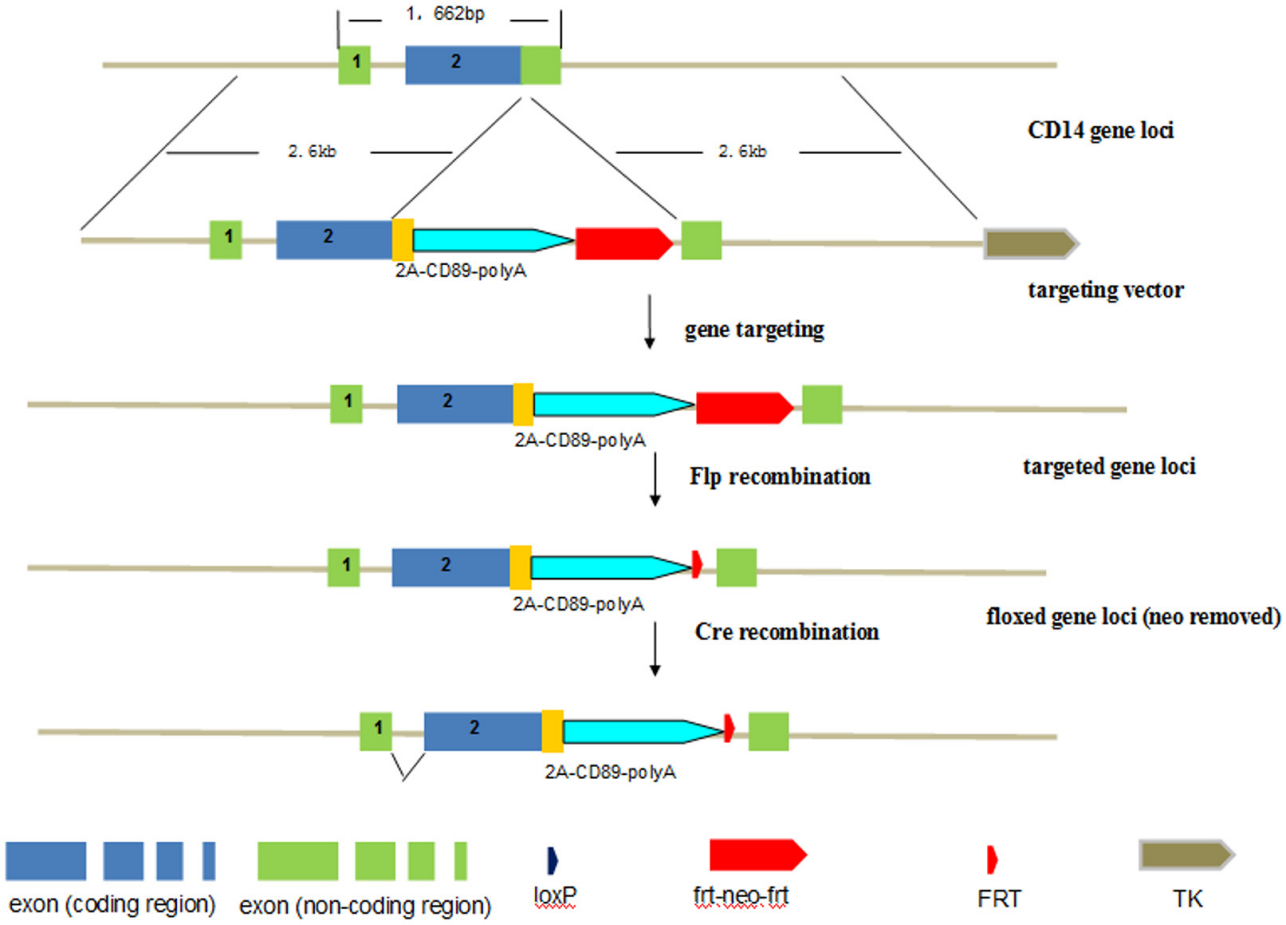
Generation of Tg mice expressing human CD89: CD14 gene knock-in strategy. The targeting vector contains 2.6kb of DNA homologous to the 5’ and 3’ sequence of the mouse CD14 gene (with blue boxes representing coding region and green boxes non-coding region), 1kb of 2A-CD89 cDNA (yellow box and blue arrow) and a *frt-neo-frt* cassette. Homologous recombination between the targeting vector and the endogenous *CD14* gene in the mouse ES cell results in the insert of the whole 2A and CD89 region. The *CD89* transgenic mice were intercrossed with FLP mice to excise the *neo* cassette.

To generate transgenic mice with CD89 expressed on the cell surface of monocyte/macrophages, a knock-in targeting vector was designed that contained a cDNA encoding the human CD89 cDNA, the 2A self-processing peptide, and a total of 5.2 kb of homology to the murine CD14 gene. 2A-CD89 was targeted to the CD14 locus using homologous recombination, resulting in the expression of human CD89 under the control of the mouse endogenous CD14 promoter (Fig 1). Homologous recombinant ES cells were micro-injected into C57BL/6 blastocysts. The resulting chimeric mice were crossed with C57BL/6 mice and a stable line was established in C57BL/6 background. To confirm expression of CD89 using this experimental strategy, CD89 expression on monocytes/macrophages was evaluated.

**Fig 2:**
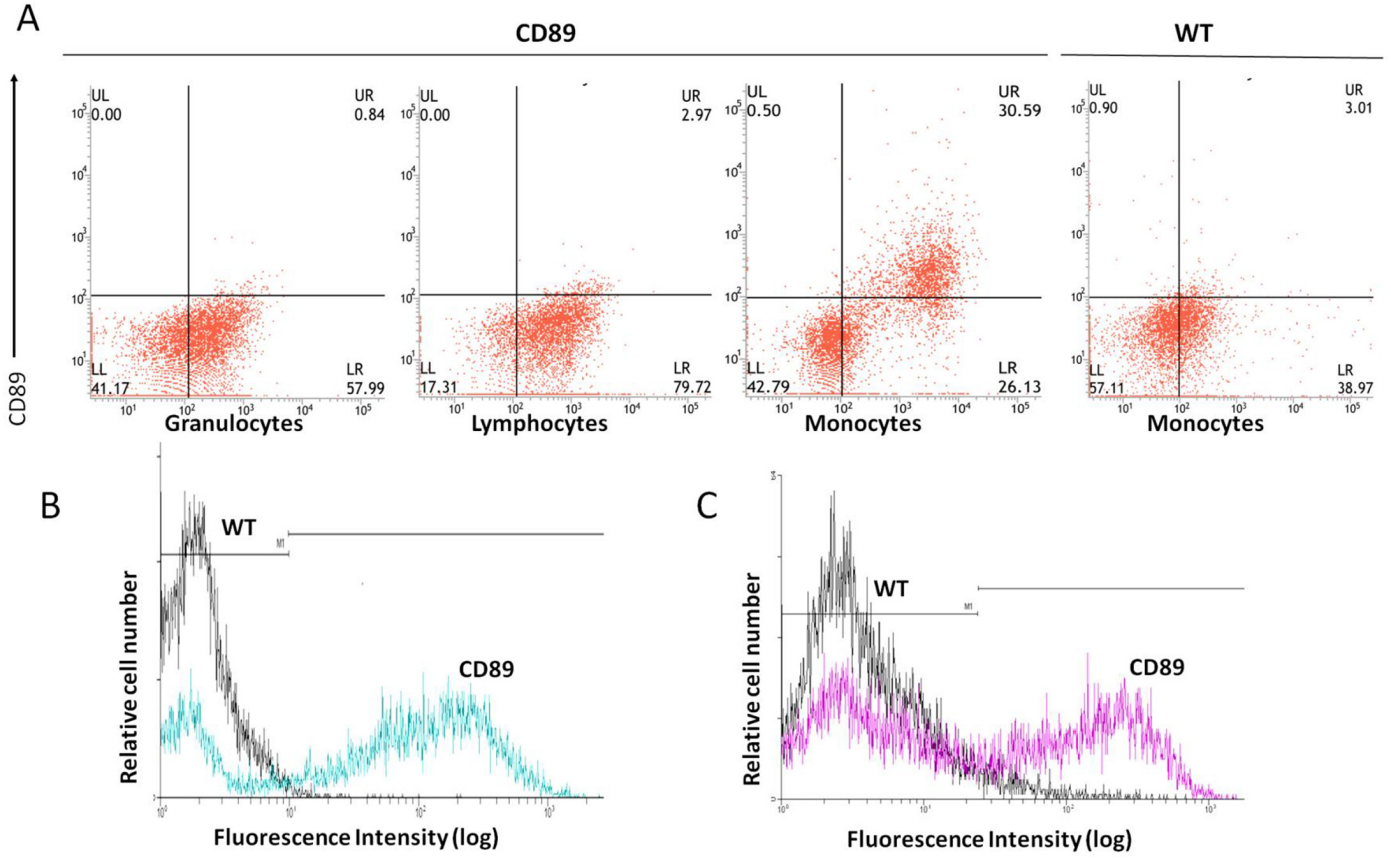
CD89 expression on the surface of monocytes/macrophages of CD89 transgenic mice. (A) Flow cytometric analysis of CD89 surface expression on mouse blood cells. Cells of non-transgenic and transgenic mice were stained with anti-CD89-FITC. Cells were stained with Gr-1-PE, CD45/B220-PE or Ly6C-PE to identify granulocytes, lymphocytes and monocytes/macrophages, respectively. Experiments shown are representative of at least three independent experiments, yielding essentially identical results. (B, C) CD89 Expression of bone marrow monocytes (B) and peritoneal macrophages (C) in Tg mice and WT mice, detected by anti-CD89-FITC mAb.

**Fig 3:**
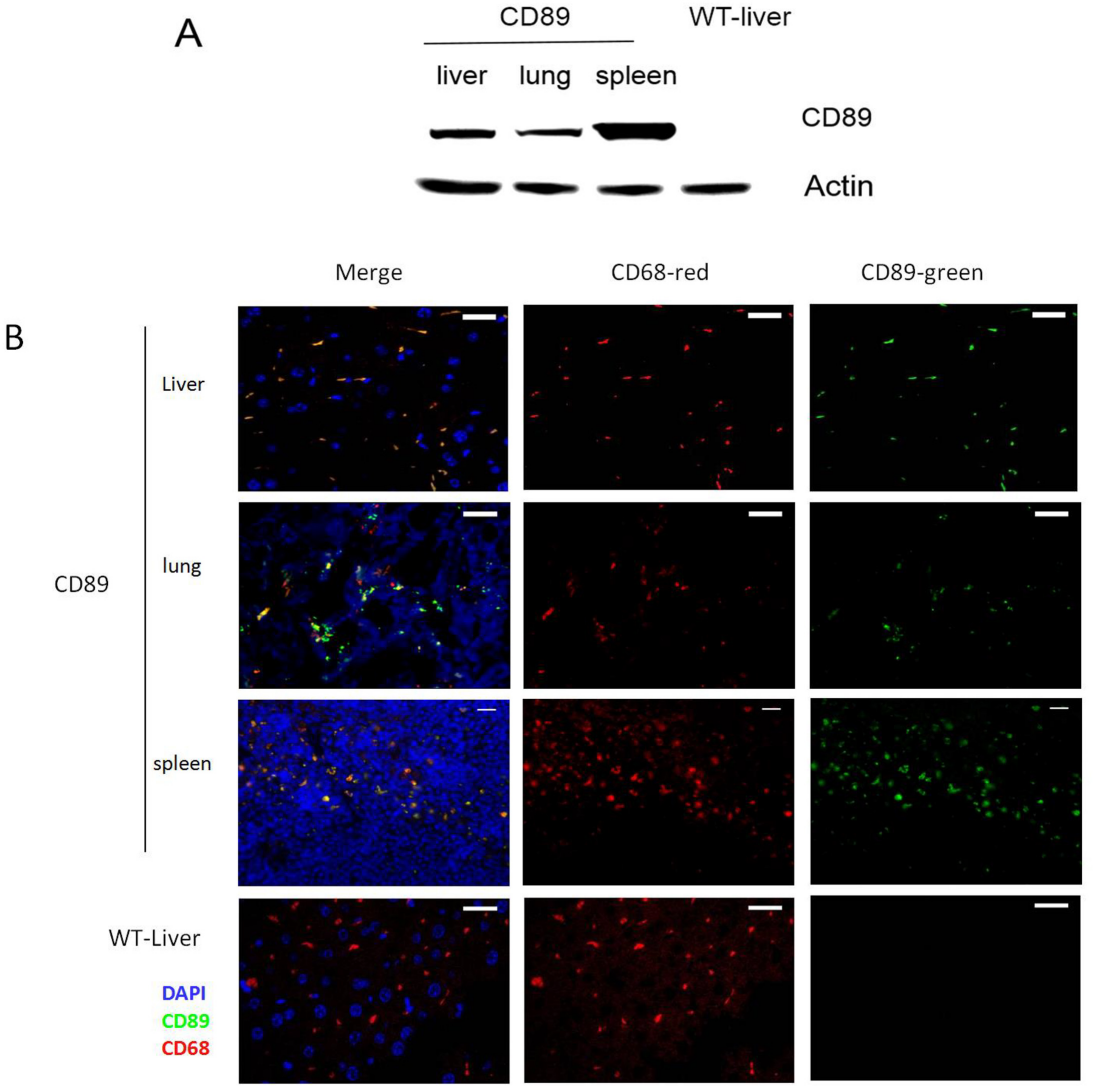
CD89 expression in the tissues of transgenic mice. (A)Western blot analysis of CD89 transgenic mice: Protein levels of the CD89 were measured in lysates of livers, lungs and spleens from CD89 transgenic mice and littermate WT mice. (B) Double immunofluorescent staining for CD68 (red) and CD89 (green) in frozen sections of liver (Kupffer cells), lung (dust cells) and spleen macrophages from CD89 Tg mice versus liver from WT mice; Cell nuclei were stained with DAPI (blue). Bar:10μm.

To this end, cells at different stages of development of the mononuclear phagocyte system were isolated from C57BL/6-Tg mice and wild type (WT) mice aged 24w. Cell types investigated included bone marrow macrophage/dendritic cell precursors, peripheral blood monocytes (Fig 2A and 2B), and macrophages derived from the peritoneal cavity (Fig 2C) and substantial CD89-specific expression in transgenic mice was observed. Granulocytes and lymphocytes were also isolated from peripheral blood of C57BL/6-Tg mice and WT mice, and no CD89 expression was detected (Fig 2A).Next, we analyzed the CD89 expression of tissue macrophages. To this end, livers, lungs, and spleens from C57BL/6-Tg mice and WT mice were lysed and probed for CD89 immunoreactivity on Western blot. Clear CD89 protein expression was found in all tissues of the Tg mice (Fig 3A). Double staining for both CD89 and the mouse macrophage marker CD68 on frozen sections from Tg mice confirmed that this expression was associated with macrophages from liver (Kupffer cells), spleen, and lung (dust cells), respectively (Fig 3B).

**Fig 4:**
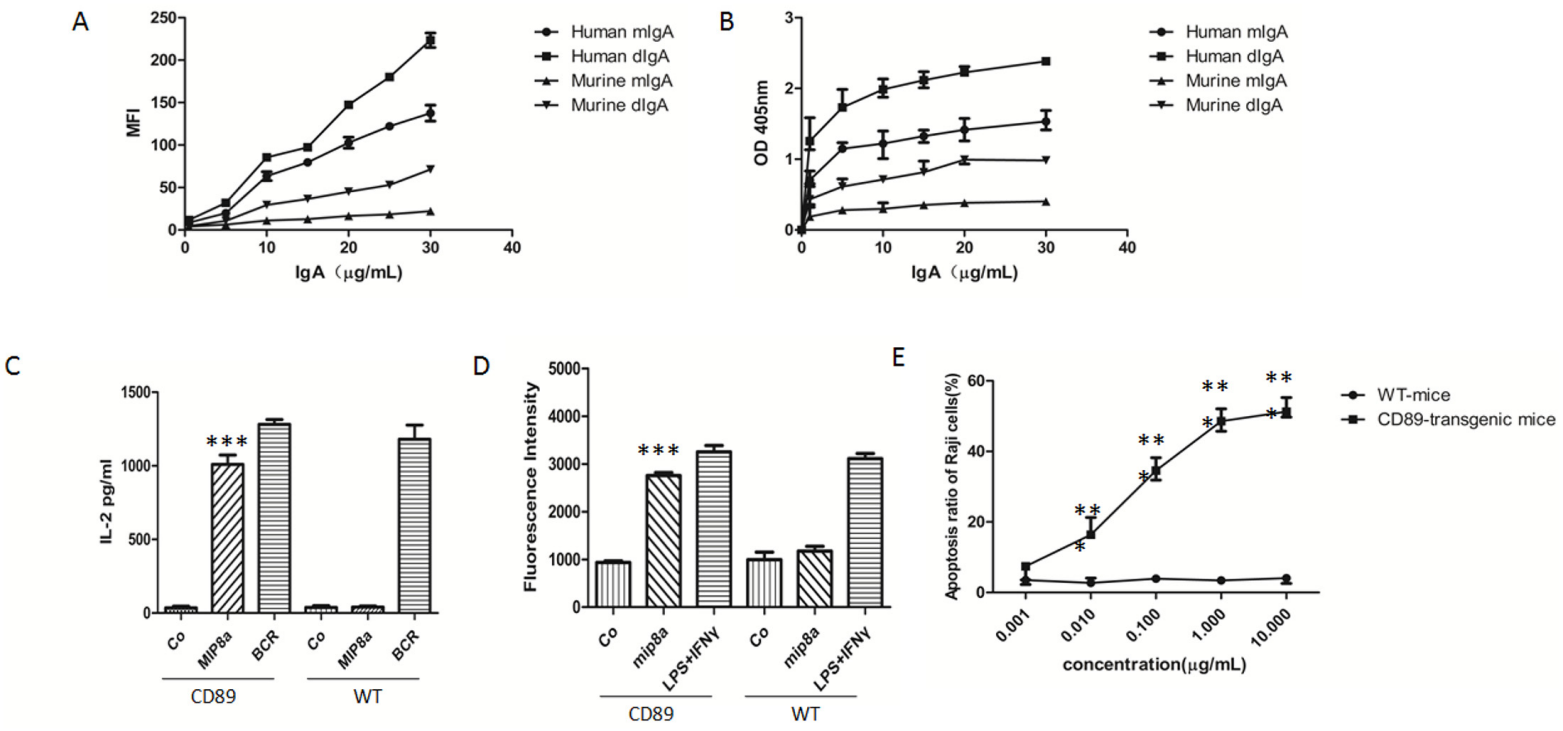
Biological functions evoked through CD89 ligationin transgenic monocytes/macrophages. (A-B) IgA binding capacity of peritoneal macrophages from CD89 Tg mice. Human dimeric IgA1 (dIgA1), monomeric IgA (mIgA) and mouse dIgA plus mIgA were used a typical dose-response curve for a FACS-binding assay data set is shown in (A) representing the mean of fluorescence intensity of IgA-CD89 on cell surface, and in (B) showing the detection of IgA-CD89 interaction by ELISA.(C) CD89 cross-linking triggered ROS production: Cells were incubated with an anti-CD89 mAb (MIP8a) and cross-linked with goat anti-mouse IgG1 to induce CD89-mediated ROS production. As a negative control, cells were solely incubated with MIP8a. As a positive control, cells were incubated with IFNγ and LPS. Intracellular ROS levels are expressed as the average DCF fluorescence intensity. ***P< 0.0001 versus values of negative co. (D) CD89-dependent IL-2 release: IL-2 production was measured with an IL-2 ELISA in supernatants after overnight stimulation following CD89 cross-linking. ***P< 0.0001 versus values of negative co. (E) ADCC mediated by CD89 on mice monocytes. Monocytes of CD89 Tg mice or WT mice were incubated with Raji tumor cells and IgA mAb. LDH release from duplicates was used as a proxy measure of tumor cell lysis. ***P<0.0001 versus values of WT mice.

We next analyzed the ability of CD89 to bind purified human and mouse IgA, using macrophages derived from the peritoneal cavity of CD89-Tg mice. Cells were incubated with increasing concentrations of human dIgA and mIgA as well as mouse dIgA and mIgA. A typical standard curve for a FACS-binding assay is shown in Fig 4A. As expected, specific binding of dimeric human and mouse IgA, respectively, by the transgenic product was observed, while no specific binding of monomeric mouse IgA was detected. ELISAs were also used to detect IgA-CD89 interaction. Increasing concentrations of human or mouse mIgA and dIgA were coated onto ELISA plates, supplemented with fixed concentrations of recombinant human CD89. The IgA-CD89 interaction was detected with CD89 antibodies. As shown in Fig 4A, bound CD89 was detected in wells coated with human IgA and mouse dIgA (Fig 4B). Thus transgene expression was appropriate and subsequent experiments were initiated to address its functionality in the murine context.

### Human CD89 Interaction with the FcRγ Chain Signaling Molecule

CD89 is a transmembrane glycoprotein lacking the capacity for autonomous signal transduction. However, binding with FcRγ chains that contain an ITAM signaling motif, and subsequent IgA binding to the CD89 –FcRγ complex trigger phagocytosis, release of inflammatory cytokines, oxidative burst, and antibody-dependent cell-mediated cytotoxicity[26]. To determine the human FcαRI expression, we measured the production of reactive oxygen species (ROS) using Fab fragments of anti-CD89 mAb (MIP8a) and secondary goat anti-mouse IgG1 to trigger cross-linking. The CD89 expression triggered ROS production following cross-linking, whereas application of secondary goat anti-mouse IgG1 alone or the use of macrophages derived from WT mice did not (Fig 4D). Macrophages from transgenic animals respond to CD89 cross-linking with IL-2 production, another canonical response of IgA binding to the CD89 –FcRγ complex[27]. As shown in Fig 4C, macrophages of Tg mice are capable of producing significant levels of IL-2 following CD89 cross-linking. In contrast, macrophages of WT mice failed to produce IL-2 upon incubation with the cross-linking complex. Furthermore, no IL-2 release was measured using an isotype control Ab (Co), whereas activation of the B cell receptor resulted in normal IL-2 production in parallel experiments. A final confirmation that our transgene was fully functional in the murine context was obtained from experiments demonstrating the ability of CD89 to trigger ADCC. To this end, the lethal potential of mouse monocytes against CD20-expressing Raji tumor cells was tested. Cells of Tg mice were capable of lysing tumor cells even in the presence of low concentrations (0.1 μg/mL) of anti-CD20 IgA antibody. No tumor cell lysis was observed using blood of WT mice (Fig 4E). We concluded that CD89 expression in our experimental system was specific and functional and enables assessment of the effect of forced expression of human CD89 in the monocyte/macrophage compartment and its subsequent effects on glomerular IgA complex deposition.

### Kidney Functional Parameters of Mice Expressing CD89

**Fig 5:**
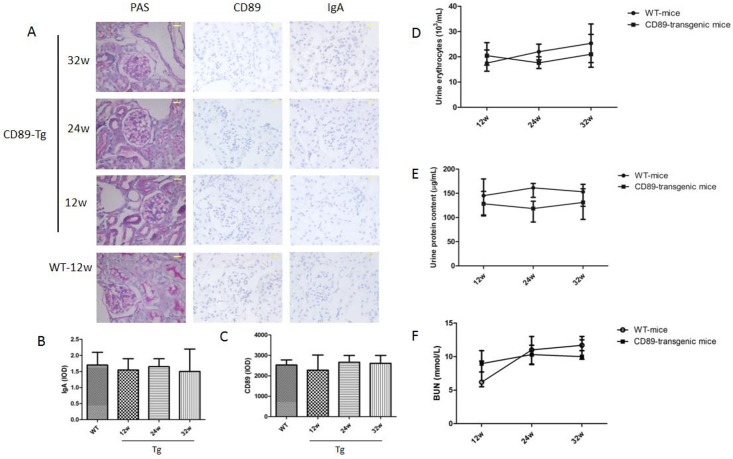
CD89 transgenic mice do not develop IgAN. (A) From the left to the right: histology of kidney sections after periodic acid-Schiff (PAS), IgA and CD89 staining of kidneys from CD89 Tg mice (10 mice per group) at different ages. No mesangial IgA deposits or periglomerular CD89 cells were seen in kidneys from CD89 Tg mice; Bar: 25μm. (B and C) The IgA and CD89 IOD were quantified in 20 randomly chosen fields for each mouse at 400 magnification. n = 4 mice *per* group; (D and E) Hematuria (×1000 red cells/mL; D) and urine protein (μg/mL) were measured in the urine of 12 to 32-wk-old WT, CD89 Tg mice (7–14 mice per group). No difference was detected between WT and CD89 Tg mice. (F) BUN levels in CD89 Tg and WT mice.

Previous results of the role of CD89 in IgAN have been partially contradictory. A transgenic approach using untargeted human CD11b promoter demonstrated IgAN with macrophage infiltration in damaged glomeruli at 12 weeks of age[21] but the specificity of this approach remains untested. Therefore, we investigated our transgenic kidneys displaying specific and functional expression of CD89 in the monocyte/macrophage compartment for renal damage. Importantly, kidneys from our C57BL/6-Tg mice showed no evidence of mesangial IgA deposition, at least until 72 weeks of age (S2 Fig). Furthermore, employing a sensitive immunohistochemical approach, no detectable deposition of IgA in kidneys from CD89 Tg mice aged 12 to 32 weeks was detected (Fig 5A and 5B). Similarly, probing for IgA deposition using an immunofluorescent approach also failed (S2 Fig). Histological analysis showed no sign of glomerular damage or interstitial tissue injury including glomerular hypertrophy or mesangial matrix expansion. No clear evidence of mesangial deposition of sCD89 in the intra- and periglomerular regions surrounding the Bowman space of the Tg mice kidneys was available (Fig 5C). In addition, no marked infiltration of macrophages was observed in the glomeruli or the intracellular space of renal tubule cells, when assessed using staining for CD89. Finally, CD89 Tg mice also presented no features of altered kidney function such as proteinuria, hematuria or increased BUN levels in the sequencing experiments (Fig 5D–5F). We concluded that CD89 expression does not confer IgAN-like features in mice.

### CD89-Mediated Clearance of IgA in Transgenic Mice

**Fig 6:**
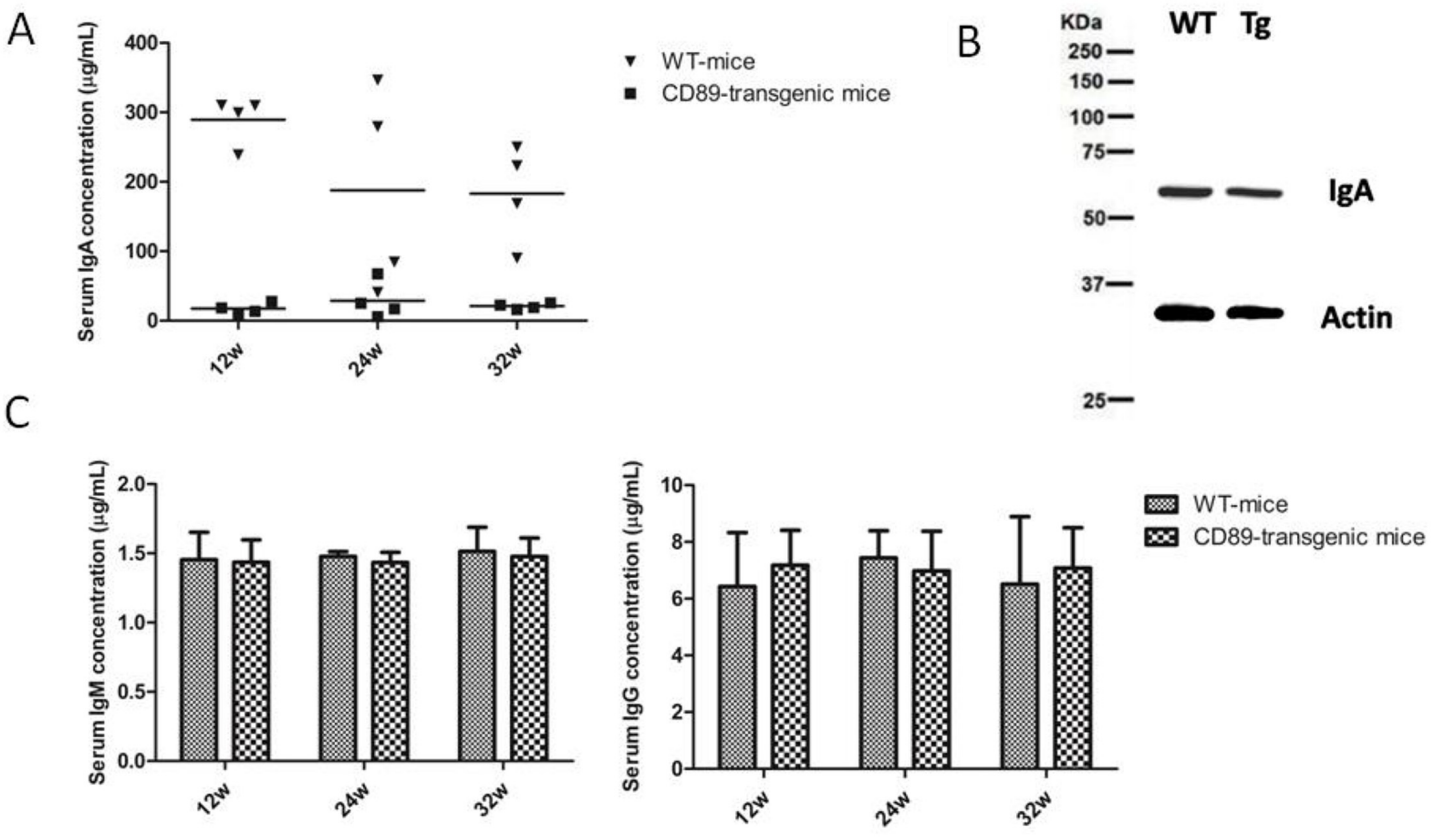
Serum IgA levels in CD89 transgenic mice were substantially decreased while IgA production by B lymphocytes was unaffected. (A) Decreased serum IgA concentration in CD89 C57BL/6-Tg mice compared with WT mice. Serum from 12-, 24-, and 32-wk-old CD89 Tg mice (n = 4) and their littermate controls (WT; n = 4) was tested with a sandwich ELISA using a commercial mouse IgA detection kit. (B) There was no significant difference between the IgA expression of blood lymphocytes in CD89 C57BL/6-Tg mice compared with WT mice. Blood lymphocytes from 24-wk-old CD89 Tg mice and their littermate controls (WT) were isolated and lysed, and analyzed using Western blot. (C-D) Serum IgM (C) and IgG (D) concentrations from CD89 C57BL/6-Tg mice were unaffected when compared with WT mice. Serum from 12-, 24-, and 32-wk-old CD89 Tg mice (n = 4) and their littermate controls (WT, n = 4) was tested with a sandwich ELISA using mouse commercial IgM and IgG detection kits.

**Fig 7:**
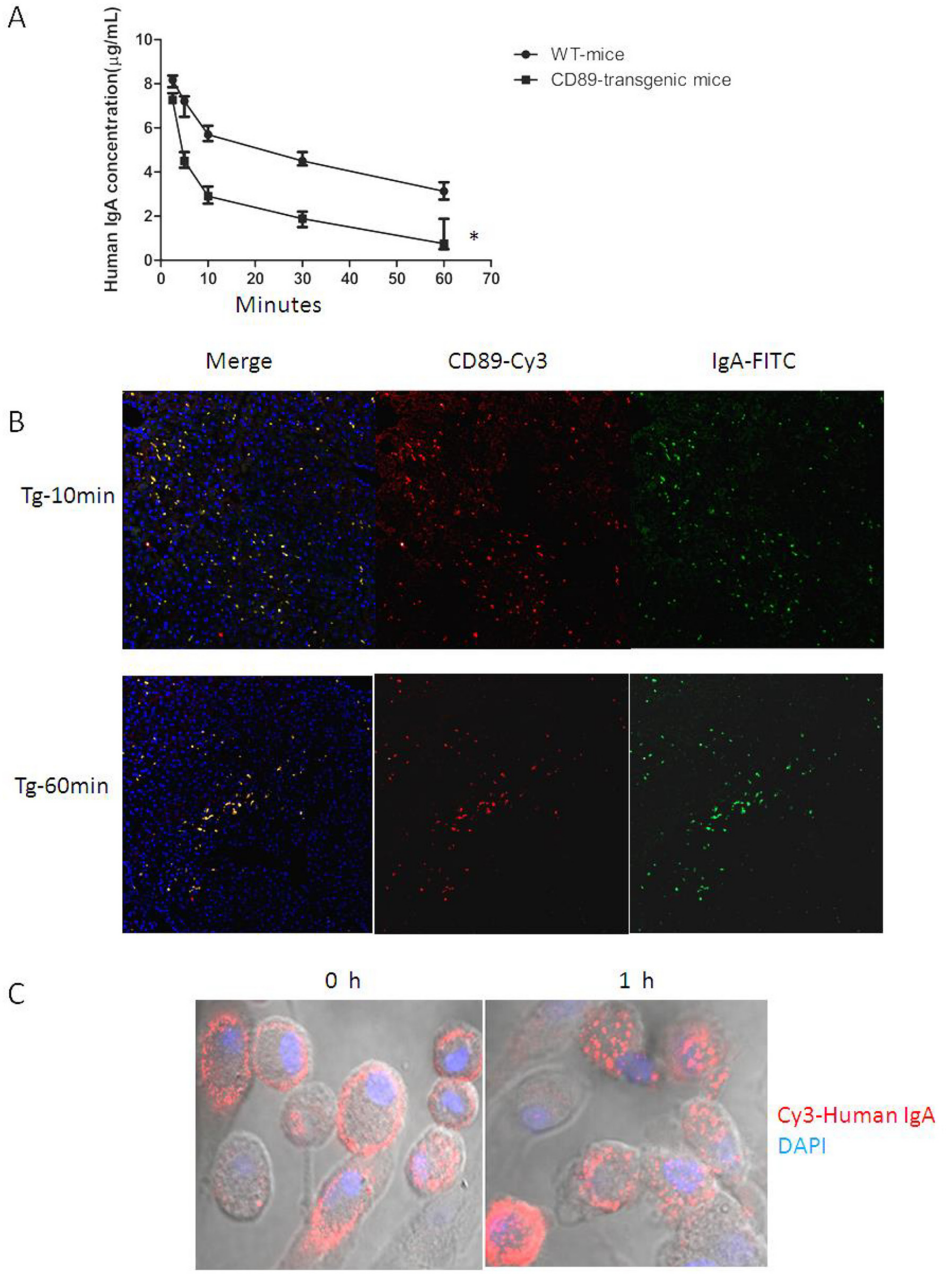
IgA clearance induced by CD89. (A) Enhanced efficiency of a 60-min serum clearance challenge by 10 μg human mIgA1 in C57BL/6-Tg mice. A total of 10 μg of human mIgA1 was injected into the tail vein of C57BL/6-Tg or WT mice (n = 5 per group). At the indicated times post-injection, sera were obtained and IgA levels were determined. IgA cleared more quickly in Tg mice(*). (B) IgA clearance by CD89 on Kupffer cells of CD89 transgenic mice. A total of 10 μg of FITC-labeled human mIgA was injected into the tail vein of C57BL/6-Tg mice (n = 5 per group). Mice were sacrificed after 10 min or 1 h, livers were stained with rabbit anti–human CD89 mAb and Cy3-goat anti-rabbit IgG, as indicated. (C) IgA clearance by CD89 on Kupffer cells from CD89 transgenic mice analyzed *in vitro*. Cy3-labeled human mIgA (1 μg/mL) was incubated with Kupffer cells from CD89 transgenic mice for 1h at 37°C.

In the transgenic mice generated, the IgA concentration in the serum of CD89 transgenic mice decreased 10-fold compared with IgA levels in WT mice (Fig 6A).Therefore, we compared the IgA levels produced by blood B lymphocytes in CD89 transgenic and WT mice using Western blot. The results revealed no difference in IgA production between the Tg and WT mice. Furthermore, when we examined serum IgG and IgM concentrations in CD89 transgenic and WT mice, no differences were detected (Fig 6C and 6D). Thus, blood B lymphocyte function *per se* was not affected in CD89 transgenic mice. The differences in serum IgA concentration between transgenic and control mice were attributed to altered clearance of IgA from the circulation consistent with the blood clearance of 10 μg human mIgA1 injected into the tail vein of C57BL/6-Tg mice. At periodic intervals after injection, blood was collected and the injected protein remaining in circulation was determined. Although human mIgA1 was cleared rapidly in both Tg and WT mice, injected IgA was eliminated more rapidly from the circulation in Tg mice than in WT mice (Fig 7A). Thus, the results suggest that IgA elimination was faster in CD89 Tg mice compared to WT mice.

Earlier published data suggested that IgA complexes endocytosed via CD89 alone were subject to recycling, while those internalized via CD89/γ2 heterodimers were degraded and sorted for antigen presentation[27].To test whether the IgA clearance was mediated by CD89/γ2 heterodimers in monocytes in our experimental system as well, we injected FITC-labeled human IgA into the tail vein of C57BL/6-Tg mice. At periodic intervals after injection, mice were sacrificed and livers were stained with rabbit anti–human CD89 mAb and Cy3-goat anti-rabbit IgG. Clear binding of FITC-IgA to CD89 positive cells (Cy3) was evident (Fig 7B). The concentration of the remaining FITC-IgA decreased at 1h than at 10 min after injection, which indicated elimination of IgA via CD89 expression on Kupffer cells in the liver of these animals. This finding was confirmed experimentally for IgA clearance by Kupffer cells isolated from CD89 transgenic mice *in vitro*. Cy3-labeled human mIgA (1 μg/mL) was incubated with Kupffer cells from CD89 transgenic mice. The cells were mounted on coverslips, permeabilized, stained, and subjected to confocal microscopy. Clear IgA endocytosis was observed after 1h of incubation at 37°C (Fig 7C). Thus, Kupffer cell CD89 clears circulatory IgA.

### Impaired Elimination of IgA in IgAN Patients via CD89

**Fig 8:**
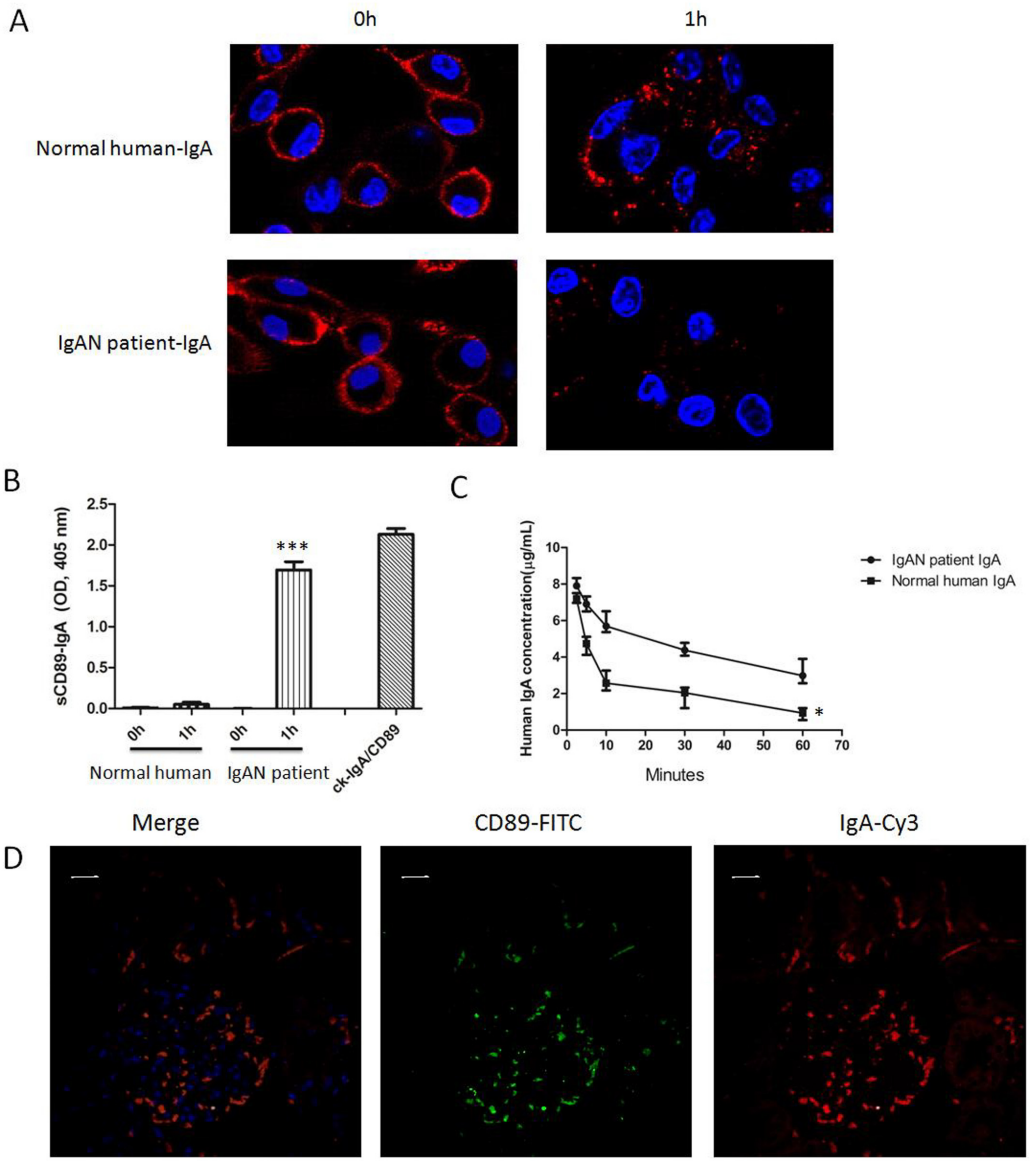
Decreased clearance of IgAN patient IgA by CD89-expressing Kupffer cells. (A) IgA, purified from a normal donor and from a patient with IgAN, was stained with Cy3-anti-human IgA Ab, and subsequently incubated with Kupffer cells from CD89 transgenic mice at a concentration of 1 μg/mL and a temperature of 37°C. After 1-h-incubation, intracellular vesicles containing IgA were diminished when IgAN patient-derived IgA endocytosis was assessed and compared with normal human IgA. (B) sCD89-IgA complexes in cell supernatant of (A) were detected by ELISA using A3 and anti-mouse IgA mAb, using 1 μg/mL human IgA binding to 5 μg/mL recCD89 as an internal control. The sCD89-IgA complexes were detected in the Kupffer cell supernatant after 1h of incubation with either control IgA or patient IgA. (C) Decreased clearance of 10 μg IgAN patient-derived IgA in C57BL/6-Tg mice when compared to normal human IgA. A total of 10 μg of each of the proteins was injected into the tail vein of C57BL/6-Tg (n = 5 per group). At the indicated times after injection, sera were obtained and IgA levels were determined. IgA from IgAN patients was cleared less quickly in Tg mice compared with normal IgA. (D) Induction of mesangial IgA/sCD89 deposits by injecting patient IgA into 6-wk-old C57BL/6-Tg mice. Double staining by anti-human IgA and anti-CD89 in kidney 48 h after constant injection of patient IgA into C57BL/6-Tg mice(n = 4) is shown. Bar = 10μm.

Abnormal interaction between IgA and CD89 in IgAN patients impairs IgA clearance and contributes to IgAN pathogenesis. To test this hypothesis directly, we investigated the clearance of IgAN patient-derived IgA by Kupffer cells CD89 *in vitro*. IgA was purified from a normal donor and from a patient with IgAN, and complexed with Cy3-labeled anti-human IgA. Proteins were incubated with Kupffer cells isolated from CD89 transgenic mice at a final concentration of 1μg/mL at 37°C. After 1h of incubation, cells were analyzed by confocal microscopy. Compared with normal human IgA, with endocytosis, intracellular vesicles containing IgAN patient IgA were undetectable (Fig 8A). It has been reported the IgA binding to CD89 induces shedding of sCD89 and formation of sCD89-IgA complexes[28]. To determine the importance of this phenomenon in our experimental setup, we established the levels of sCD89-IgA complexes in the cell supernatant before and after incubation of transgenic mouse-derived Kupffer with IgA. ELISAs were performed using A3 and anti-human IgA mAb, whereas 1 μg/mL human IgA binding to excess recombinant CD89 (5 μg/mL) served as an internal control. The sCD89-IgA complexes were easily detected in the cell supernatant after 1h incubation of patient IgA with Kupffer cells(Fig 8B), whereas no sCD89-IgA complexes were detected when normal human IgA was employed. Thus, IgA from healthy individuals is cleared by Kupffer cell CD89, unlike in IgAN patients.

We further compared the clearance of IgA from 10 normal donors and 10 patients with IgAN, using an *in vivo* assay. We injected 10 μg of the respective IgA preparations into the tail vein of C57BL/6 Tg-mice (n = 5 per group). At the post-injection times indicated, sera were obtained and the human IgA levels remaining in circulation were determined. Normal human IgA was eliminated more rapidly from the circulation than in patients with IgAN. Fig 8C shows a representative example. However, no difference in human IgA elimination was found when control human IgA and IgAN patient IgA were injected into WT mice (S3 Fig). Thus, the defective IgA clearance in IgAN requires CD89.

The injection of IgAN patient IgA has been established to provoke IgAN in SCID CD89 Tg mice [21]. To further establish the underlying mechanism, we injected purified patient IgA and normal IgA (100 μg) into 6-wk-old C57BL/6-Tg mice and WT mice. Interestingly, the patient IgA induced heavy and sustained macroscopic hematuria over a 72-h period in Tg mice, whereas normal IgA induced no microscopichematuria. No microscopichematuria was observed in WT mice (S4 Fig). As shown in Fig 8D, heavy mesangial mass sedimentation and human IgA particle deposition was observed after 48h injection of patient IgA in the context of forced CD89 expression, whereas no such deposition was found in the kidneys of Tg mice injected with normal IgA or in WT mice injected with patient IgA(S4 Fig). Thus the interaction of patient IgA with CD89 induces IgAN in our Tg mice in a C57BL/6 background, in apparent agreement with previous reports on experimental IgAN.

## Discussion

In this paper the relation between CD89 and IgA clearance especially in relation to IgAN, was investigated. Previous data reported possible CD89-mediated IgA clearance, especially via Kupffer cells[18–20,29]. However, the mechanism of IgA elimination from the circulation via CD89 remained unclear and no direct evidence supporting the role of Kupffer cells in human IgA clearance was provided. A major hurdle was the lack of an experimental system for direct study of such CD89-mediated IgA clearance. Many experimental animals, including rats and chimpanzees, have FcαRI equivalent receptors in their genome, but are not amenable to experimental investigation[30,31].

The power of transgenic technology, however, allows expression of human genes in a murine setting. Two classic CD89 transgenic mice have been reported in the literature. In a mouse model generated by Marjolein van Egmond, the CD89 expression was restricted to neutrophils and Kuppfer cells. However, the induction of CD89 expression in Kupffer cells in this model depended on exogenous G-CSF [18,32], complicating the analysis of CD89 function. A second CD89-C57BL/6-Tg transgenic mouse model, however, was characterized by mesangial IgA deposition and hematuria controlled by a human CD11b promotor, and IgAN developed at an age of 12 weeks[21], suggesting a prominent role for CD89 in IgAN development. This model assumed that the IgAN was induced by a robust heterologous CD89 expression. In our study, we investigated the effect of CD89 expression using a murine CD14 promoter. CD11b and CD14 are both excellent markers for monocytes and macrophages[33,34]. Previous studies demonstrated that mouse monocytes universally express CD11b in conjunction with less consistent expression of CD14 [35]. CD89 expression was demonstrated on the monocytes and macrophage compartment of our transgenic mice. The transgenic receptor binds both human and dimeric mouse IgA. It is capable of triggering signaling through the FcRγ chain and is therefore, fully functional. Thus, this model allows a direct analysis of the function of human CD89 in a murine model. As mice are characterized by small circulatory volume, murine models enable testing of purified human proteins, even in limited amounts. Therefore, the murine model facilitates the study of interaction between human CD89 and patient-derived serum components.

Here, we used an appropriate promotor to demonstrate that forced expression of human CD89 in the myeloid lineage is possible without concomitant IgA nephropathy, which was opposite to observation in a previous study [21]. This discrepancy can be explained from lower transgenic CD89 expression on monocytes and macrophages as that driven by hCD11. In conjunction with the data presented by Launay et al our data strongly suggest that in experimental IgAN the mouse IgA deposits are clearly dependent of the levels of transgenic CD89 expression achieved. It has been demonstrated that mouse IgA binds to hCD89 with relatively low efficiency [26,36,37] and this might be one explanation for the absence of detection of mouse IgA deposits in our model. Human IgA1 KI crossed with hCD11b promoter-CD89 Tg developed IgAN at an earlier age of 6 weeks, consistent with the notion that human CD89 preferentially binds to human IgA as compared to mouse IgA[37]. Furthermore, different mouse strains may have different susceptibility to glomerulonephritis, also explaining variance in IgAN manifestions between different models. Our model, without spontaneous IgAN but prone to develop the disease following injection of patient IgA allowed us to perform mechanistic studies and in particular to elucidate the role of the Kupffer cell in IgAN pathogenesis.

Interestingly, the serum IgA concentration of transgenic mice was decreased 10-fold compared with IgA levels in WT controls, despite normal levels of IgA production in murine B cells revealed by Western blot and IgA-specific sera (Fig 6A and 6B). Therefore, human CD89 mediates IgA clearance from the circulation. Direct evidence supporting this finding was further obtained in experiments in which exogenous human IgA was injected into the tail vein of C57BL/6 Tg or WT mice. The injected proteins were cleared more quickly from the circulation in Tg mice (Fig 7A). Hence, CD89 represents a bona-fide mediator of IgA clearance from the circulation.

IgA clearance mediated by ASGPR expression in hepatocytes via O-linked sugar moieties on IgA molecules has been unequivocally established. In IgAN, it has been postulated that the macromolecular IgA-ICs containing IgA1 lacking galactose residues on the O-linked sugars escape hepatic clearance by failing to bind ASGPR and thus induce subsequent deposition of IgA complexes in the mesangium[38–40]. However, ASGPR appears to mediate the clearance of IgA2 predominantly, and seems to play a relatively minor role in the clearance of IgA1[13], suggesting the possibility of other mechanisms underlying IgA1 elimination from the circulation. Here, we found that CD89 plays an important role in IgA1 clearance. Injected human mIgA1 is eliminated more rapidly from the circulation in Tg mice than in WT mice (Fig 7A). Furthermore, binding of injected FITC-IgA1 to CD89 positive Kupffer cells was observed in CD89 transgenic mice, which also triggered subsequent destruction of the injected FITC-IgA1, as confirmed *in vitro* using isolated Kupffer cells from Tg mice. *In toto*, CD89 appears a potent mediator of IgA1 clearance when transgenically expressed in liver cells.

Previous studies showed that the soluble Fc-α receptor (FcαR/sCD89) was involved in IgA complex formation and IgAN, with different sCD89 isoforms associated with either pathogenic or protective actions[41]. IgA complexes containing 50- to 70-kDa sCD89 are exclusively detected in IgAN patients, and the concomitant downregulation of CD89 expression in monocytes and neutrophils in the blood of IgAN patients suggested enhanced shedding of the molecule, causing higher serum CD89 levels[19]. However the role of sCD89 in IgAN remains controversial. Other investigators failed to detect decreases in monocyte mCD89 expression using an indirect immunofluorescence assay[42]. Injection of human IgA receptor FcαRI/CD89 in mice fails to provoke IgAN-like symptoms[43]. Our study thus further urges for caution in interpreting the data supporting the role of sCD89 in IgAN pathogenesis.

In the present study, the formation of IgA-CD89 complexes after IgA binding-induced CD89 shedding was detected. IgA purified from a normal donor and from a patient with IgAN, and incubated with Kupffer cells from CD89 Tg mice resulted in the appearance of sCD89-IgA complexes in the cell supernatant (Fig 8B), but only using patient-derived IgA. Increased binding of patient IgA to CD89 has been reported compared with normal IgA, indicating that abnormal IgA played a role in CD89 shedding. CD89 binds to IgA between the CH2 and CH3 domains. The disorders of CH2 glycosylation may be associated with CD89-IgA interaction. We also observed impaired clearance of IgAN patient-derived IgA by Kupffer cell CD89 in *in vitro* assays, when results were compared with IgA isolated from healthy controls. In apparent agreement, the intracellular vesicles containing endocytosed IgA were undetectable with IgAN patient-derived IgA, but were evident using IgA from healthy controls (Fig 8A). Apparently, IgAN-derived IgA was resistant to clearance by Kupffer cells, and induces concomitant shedding of CD89 and IgA-IC release. Apparently consistent with this notion was the observation that the clearance of IgAN patient-derived IgA was temporally reduced in CD89 Tg mice when compared with that of normal human IgA. Patient IgA induced significant mesangial human IgA deposition and IgAN features 48h following injection (Fig 8C). In contrast, no such deposition was found in the kidneys of Tg mice injected with normal IgA and WT mice injected with patient IgA, indicating that specific combination of patient IgA with CD89 is necessary for development of experimental IgAN.

In conclusion, our results reveal a cardinal role of Kupffer cells in IgA clearance mediated by forced CD89 expression in mice and especially IgA1. Furthermore, they reinforce the significance of the combination of patient IgA and CD89 for the development of IgAN. Thus, our Tg mice represent an excellent model to study CD89 functionality in general and IgAN in particular.

## Supporting Information

S1 Fig The positive staining of mouse dodecadactylon for mouse IgA, served as the positive control. Bar: 20μm.
(TIF)

S2 Fig From the left to the right: DAPI, CD89-FITC and IgA-Cy3 staining of kidneys from CD89 Tg mice (10 mice per group) at different ages. No mesangial IgA deposits or periglomerular CD89 cells were seen in kidneys from CD89 Tg mice.
(TIF)

S3 Fig Clearance of 10 μg IgAN patient-derived IgA andnormal human IgA in C57-WT mice. A total of 10 μg of each of the proteins was injected into the tail vein of C57 WT (n = 5 per group). At the indicated times after injection, sera were obtained and IgA levels were determined. No difference in human IgA elimination was found when control human IgA and IgAN patient IgA were injected into WT mice.
(TIF)

S4 Fig Related to Fig 8. (A) Erythrocyte counts at the times indicated after injection of purified patient IgA andnormal IgA (100 μg) into 6-wk-old C57-Tg miceand C57-WT mice(n = 5 per group). (B) Double staining by anti-human IgA(Red) and anti-CD89 (Green) in kidney 48 h after constant injection of patient or normal IgA into C57-Tg mice or C57-WT mice (n = 4) is shown. Bar = 10μm. Patient IgA could not induce IgA deposition without CD89. And normal IgA could not induce IgA deposition in both C57-Tg mice and C57-WT mice.
(TIF)

S1 Table Clinical characteristics and laboratory data of the patients with IgAN and healthy controls at the time point of serum IgA measurement. (TIF)
