# Supplementary figures and images for: Critical Role of Kupffer Cell CD89 Expression in Experimental IgA Nephropathy
Source: PLoS One. 2016 Jul 20;11(7):e0159426. doi: 10.1371/journal.pone.0159426 (PMC4954728; doi:10.1371/journal.pone.0159426)

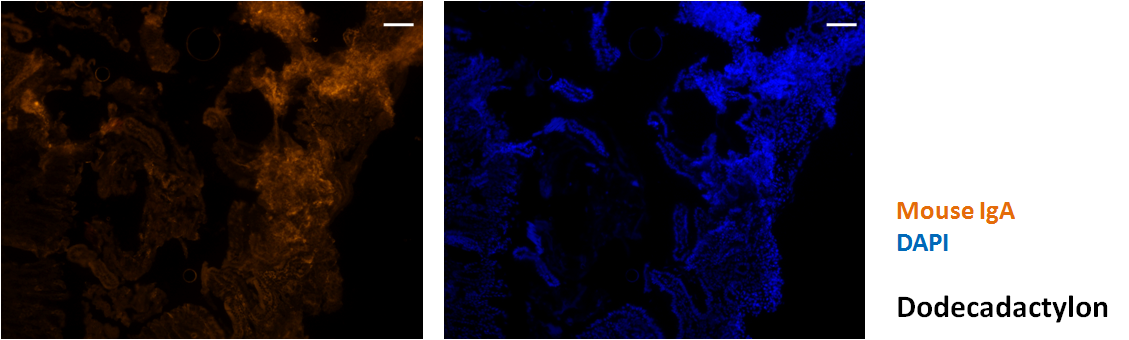

Supplement: S1 Fig — Bar: 20μm. (TIF) [file pone.0159426.s001.tif]

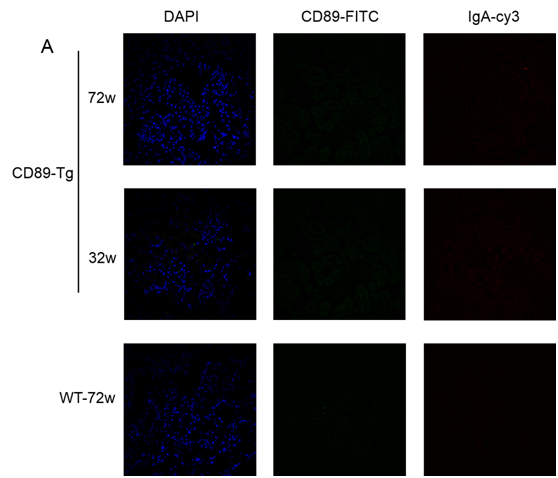

Supplement: S2 Fig — No mesangial IgA deposits or periglomerular CD89 cells were seen in kidneys from CD89 Tg mice. (TIF) [file pone.0159426.s002.tif]

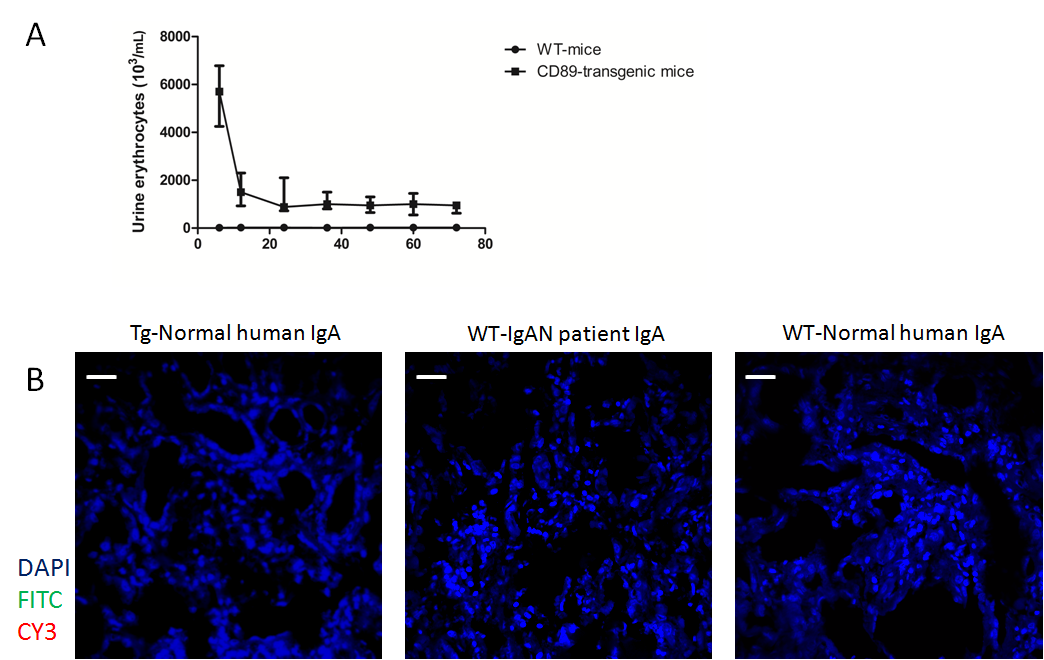

Supplement: S4 Fig — (A) Erythrocyte counts at the times indicated after injection of purified patient IgA andnormal IgA (100 μg) into 6-wk-old C57-Tg miceand C57-WT mice(n = 5 per group). (B) Double staining by anti-human IgA(Red) and anti-CD89 (Green) in kidney 48 h after constant injection of patient or normal IgA into C57-Tg mice or C57-WT mice (n = 4) is shown. Bar = 10μm. Patient IgA could not induce IgA deposition without CD89. And normal IgA could not induce IgA deposition in both C57-Tg mice and C57-WT mice. (TIF) [file pone.0159426.s004.tif]

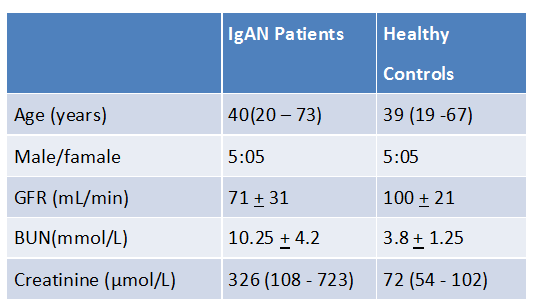

Supplement: S1 Table — (TIF) [file pone.0159426.s005.tif]
